# Evolution of Emergency Airway Management From Anatomical Prediction to a Systems-Based Resuscitative Approach in the Emergency Department: A Narrative Review

**DOI:** 10.7759/cureus.115046

**Published:** 2026-08-23

**Authors:** Anagani Hrushikesh, Chinnam Vishnupriya, Chetla Rakesh, Anusree K, Dhanasekaran B S, Gireesh Kumar

**Affiliations:** 1 Emergency Medicine, Amrita Institute of Medical Sciences, Kochi, IND; 2 Biostatistician, Amrita Institute of Medical Sciences, Kochi, IND

**Keywords:** airway management, emergency department, first-pass success, heaven criteria, human factors, narrative review, peri-intubation cardiac arrest, physiologically difficult airway, point-of-care ultrasound, video laryngoscopy

## Abstract

Airway management in the emergency department (ED) is one of the most critical and time-pressured procedures in acute care medicine. Patients frequently present with severe physiological compromise, including severe hypoxia, hemodynamic instability, shock, altered mental status, trauma, or cardiac arrest, leaving clinicians with minimal preparation time and incomplete clinical information. While anatomical predictors of laryngoscopic difficulty have traditionally guided pre-intubation assessment, growing evidence suggests that the greatest threats during emergency airway management are often physiological rather than anatomical. Conditions such as profound hypoxemia, hemodynamic instability, metabolic acidosis, and severe respiratory failure significantly increase the risk of peri-intubation complications, including cardiovascular collapse and cardiac arrest, regardless of airway anatomy.

PubMed, Google Scholar, and Scopus were searched from January 2000 through December 2025 using structured Boolean search terms, supplemented by hand-searching reference lists and relevant society guidelines. Relevant studies were selected according to predefined eligibility criteria and synthesized narratively.

Among the included studies, physiological instability, particularly hypoxemia, metabolic acidosis, and circulatory shock, was consistently associated with an increased risk of peri-intubation cardiac arrest and appeared to be at least as important as bedside anatomical findings in emergency airway risk assessment. Current evidence supports a structured approach to emergency airway management that includes physiological optimization before laryngoscopy, use of the HEAVEN criteria for airway risk stratification, individualized drug selection based on the patient's hemodynamic status, structured airway bundles, video laryngoscopy as the preferred primary intubation device in appropriate emergency airway settings, and team-based cognitive tools such as the Vortex Approach. The DEVICE randomized trial demonstrated a 14.3-percentage-point improvement in first-pass success with video laryngoscopy versus direct laryngoscopy in critically ill adults. Prospective validation of these approaches in lower-resource settings, with neurological recovery rather than intubation success alone as the primary outcome, remains an important research priority.

## Introduction and background

Emergency department (ED) airway management is among the most time-sensitive and high-risk procedures in acute care medicine. The ED environment bears little similarity to an elective operating theater: patients may be agitated, uncooperative, or obtunded; oxygenation reserves are often low by the time they arrive, and hemodynamic status can deteriorate within minutes of presentation. Clinicians are required to make rapid pharmacological and procedural decisions with incomplete histories, uncertain fasting status, and no opportunity for the systematic pre-assessment that is standard practice in elective anesthesia settings. In this context, techniques such as rapid sequence intubation (RSI), delayed sequence intubation (DSI), and aggressive preoxygenation are now widely used to minimize the risk of desaturation before laryngoscopy in patients who are agitated, unresponsive, or critically hypoxic on arrival [1,2].

When airway management fails in the ED, the consequences are immediate and life-threatening. Patients who cannot maintain a patent airway even for a brief period are at risk of catastrophic outcomes, including aspiration pneumonitis, hypoxic-ischemic encephalopathy, and peri-intubation cardiac arrest (PICA) [3,4]. What makes the ED particularly dangerous is that these risks do not appear one at a time. The patient may already be in shock before the procedure starts. Their condition may be worsening rapidly. The team may be working under significant time pressure in a busy ED, where multiple critically ill patients often require simultaneous attention.

Trauma represents a distinct category of emergency airway management that needs specific attention. Maxillofacial fractures can destroy normal glottic landmarks, active bleeding from oropharyngeal injuries can reduce airway visibility within seconds, and mandatory cervical spine immobilization prevents the neck extension required for adequate glottic visualization during direct laryngoscopy. In trauma, airway adjuncts such as the bougie and the suction-assisted laryngoscopy and airway decontamination (SALAD) technique are now standard tools when the airway view is poor or contaminated [5,6]. Importantly, airway assessment and management cannot occur in isolation. In trauma resuscitation, airway management, hemorrhage control, and chest decompression often need to proceed in parallel because delays in any of these interventions may contribute to rapid clinical deterioration.

An important distinction that must be recognized rapidly at the bedside is the difference between a difficult airway and a crash airway. A difficult airway is one in which intubation is expected to be technically difficult [7,8]. Still, the patient retains enough physiological reserve to allow time for preoxygenation, drug preparation, team briefing, and a structured backup plan. A crash airway is fundamentally different. These patients are in cardiac arrest, are agonal, or are deteriorating so rapidly that any delay for pharmacological preparation is itself dangerous. In patients with cardiac arrest, the laryngoscope is picked up immediately without induction, as there is no time for physiological optimization. In patients with peri-arrest physiology or profound hemodynamic instability, however, airway management should be individualized based on the patient’s clinical condition. If direct laryngoscopy fails and bag-mask ventilation cannot maintain oxygenation and ventilation, emergency cricothyrotomy is the only remaining option. A key clinical risk is misclassifying a crash airway as a difficult airway. In patients with no physiological reserve, delaying definitive airway intervention for optimization can be harmful.

The epidemiological data on emergency airway complications make a strong case for the approach this review advocates. Major airway-related adverse events occur at substantially higher rates in the ED compared with the operating theater, a pattern documented consistently by the UK Fourth National Audit Project and the US National Emergency Airway Registry [3]. What these registries also demonstrate is that risk increases with each failed attempt: hypoxemia deepens, blood pressure falls further, and the probability of cardiac arrest rises with every additional laryngoscopy [3,4]. PICA complicates approximately 1% to 4% of emergency intubations [4]. The present review traces how emergency airway management has evolved from anatomy-based assessment toward a physiology-first approach and draws on the current evidence to offer a practical framework for improving safety and reducing preventable harm in the ED.

## Review

Search strategy and study selection

We conducted a structured narrative review of literature published between January 2000 and December 2025 in PubMed, Scopus, and Google Scholar, supplemented by hand-searching relevant guidelines and reference lists. Eligible literature was narratively synthesized to describe the evolution of emergency airway management from anatomy-based prediction toward physiology-focused and systems-based practice.

We searched three electronic databases: PubMed (National Library of Medicine), Google Scholar (Google LLC), and Scopus (Elsevier). The search covered literature published from January 2000 to December 2025. PubMed was prioritized given its comprehensive indexing of emergency medicine and critical care literature, while Google Scholar was included to capture relevant sources not indexed in standard databases. Google Scholar search results were screened based on their relevance to the review topic, and eligible studies were considered for inclusion in the narrative synthesis. In addition to electronic searching and relevant society guidance, including the 2022 American Society of Anesthesiologists (ASA) guideline, the 2018 Difficult Airway Society (DAS) guideline was also considered [7,8]. This hand-searching identified three additional studies that electronic searching had not captured.

A structured search strategy based on common Boolean concepts was applied across the three databases, combining terms related to the clinical setting, airway management, difficult airway assessment, physiological risk, and relevant airway interventions. Database-specific modifications to the search syntax were made where necessary, particularly for Google Scholar, to accommodate differences in search functionality while maintaining the same core search concepts across all databases. The search terms included combinations of “emergency department,” “emergency medicine,” “emergency airway,” “prehospital,” “airway management,” “intubation,” “rapid sequence intubation,” “RSI,” “video laryngoscopy,” “direct laryngoscopy,” “difficult airway,” “first-pass success,” “peri-intubation cardiac arrest,” “physiologically difficult airway,” “HEAVEN criteria,” “LEMON criteria,” “point-of-care ultrasound,” “cricothyrotomy,” “supraglottic airway,” “preoxygenation,” “apneic oxygenation,” “human factors,” “Vortex approach,” “bougie,” and “SALAD.”

Inclusion and exclusion criteria were defined before the search was conducted. We included studies involving adult and pediatric patients requiring emergency airway management in ED or prehospital settings. Eligible interventions included emergency intubation, RSI, video laryngoscopy, supraglottic airway insertion, and physiological preoptimization strategies. Primary outcomes of interest were first-pass intubation success rate, PICA, and procedure-related adverse events. Eligible study designs included RCTs, systematic reviews, meta-analyses, observational studies, national audits, consensus guidelines, and methodologically rigorous narrative reviews. We excluded case reports involving fewer than five patients, animal studies, non-English publications, and studies confined exclusively to elective surgical environments.

Database searching identified 970 records (PubMed: 350; Scopus: 420; Google Scholar: 200 relevance-ranked results screened). After removal of 100 duplicates, 870 unique records were screened by title and abstract. Combined with the three additional records identified through hand-searching, 100 records underwent full-text assessment, of which 72 were excluded. Twenty-eight studies met the eligibility criteria and were included in the final synthesis. Priority was given to relevant and higher-quality evidence, including randomized controlled trials, systematic reviews and meta-analyses, observational studies, national audits, clinical practice guidelines, and key narrative reviews. Findings were synthesized narratively, with consideration of the methodological strengths and limitations of the available evidence.

Relevant studies identified through the database searches and manual reference screening were selected according to the predefined eligibility criteria and included in the narrative synthesis. A quantitative synthesis was not performed owing to substantial heterogeneity in study designs, populations, interventions, and outcome definitions; the findings were therefore synthesized narratively. The characteristics of the included studies are presented in Table 1.

**Table 1:** Characteristics of included studies (n=28) NMBA: neuromuscular blocking agent; CPG: clinical practice guideline; RCT: randomized controlled trial; SR: systematic review; Obs.: observational; DSI: delayed sequence intubation; ED: emergency department; NAP4: UK fourth National Audit Project; RSI: rapid sequence intubation; FPS: first-pass success; SALAD: suction-assisted laryngoscopy and airway decontamination; ASA: American Society of Anesthesiologists; VL: video laryngoscopy; DAS: Difficult Airway Society; HEAVEN: hypoxemia, extremes of size/age, anatomical abnormalities, vomiting/blood, exsanguination, neck movement; POCUS: point-of-care ultrasound; CTM: cricothyroid membrane; CO_2_: carbon dioxide; DL: direct laryngoscopy; DEVICE: direct versus video laryngoscope; NEJM: New England Journal of Medicine; CICO: cannot intubate, cannot oxygenate; eFONA: emergency front-of-neck access; OHCA: Out-of-Hospital Cardiac Arrest; NEAR: National Emergency Airway Registry; SAM: Society for Airway Management

Author, year	Design	Setting	Key focus	Main contribution
Weingart, 2011 [1]	Narrative review	ED	Preoxygenation, DSI	Introduced DSI; established aggressive preoxygenation as standard practice to reduce desaturation risk
Lentz et al., 2020 [2]	Narrative review	ED	High-risk airway diseases	Disease-specific framework for stratifying airway risk across conditions, including epiglottitis and critical asthma
Cook et al., 2011 [3]	NAP4 audit	ICU/ED (UK)	Major airway complications	ED had highest complication rates vs. OR; human factors were identified as primary contributors
Walls et al., 2011 [4]	Multicenter obs. (NEAR)	ED	8,937 intubations	RSI was dominant technique; FPS was established as key safety metric; adverse events correlate with attempt number
Driver et al., 2018 [5]	RCT	ED	Bougie vs. stylet	Bougie significantly improved first-attempt intubation success among patients with difficult airway characteristics (96% vs. 82%)
Root et al., 2020 [6]	Technical report	ED/prehospital	SALAD technique	SALAD provides a superior glottic view in contaminated airway; essential for trauma and aspiration scenarios
Apfelbaum et al., 2022 [7]	CPG (ASA)	OR/ED	Difficult airway guidelines	2022 ASA guidelines endorse VL and supraglottic airways as primary tools; mandate team briefing and rescue plan
Higgs et al., 2018 [8]	CPG (DAS)	OR/ED/ICU	Critically ill adults	DAS guidelines establish preoxygenation standards, team briefing, and stepwise rescue planning
Jaber et al., 2010 [9]	Prospective interv.	ICU	Structured intubation bundle	A formal intubation checklist significantly reduced life-threatening intubation complications in the ICU
Reed, 2005 [10]	Prospective obs.	ED	Airway scoring tools	Traditional anatomical tools have limited discriminative accuracy in emergency populations
Tan et al., 2022 [11]	Prospective obs.	ED	HEAVEN criteria	The presence of any HEAVEN criterion was associated with lower first-pass success; anatomical challenge predicted first-pass success, while hypoxemia and vomit/blood/fluid were associated with intubation complications
Kerrey et al., 2012 [12]	Retrospective cohort	Pediatric ED	Pediatric RSI outcomes	Higher frequency of failed intubation attempts and adverse effects in pediatric emergency RSI; informs pediatric-specific protocol design
Gottlieb et al., 2024 [13]	Systematic review	ED/ICU	POCUS for airway	POCUS improves CTM identification accuracy and enables reliable real-time confirmation of tube placement
Mosier et al., 2015 [14]	Review	ED/ICU	Physiologically difficult airway	Defined the physiologically difficult airway; physiological instability drives peri-intubation complications more than anatomy
Singer et al., 2022 [15]	Retrospective cohort	ED/ICU	Push-dose vasopressors	Push-dose epinephrine/phenylephrine safe and effective; supports pre-intubation hemodynamic optimization
Kraut and Madias, 2010 [16]	Review	General/ICU	Metabolic acidosis	Characterized apnea-induced CO_2_ accumulation and pH collapse in acidotic patients; provides a rationale for DSI
Kornas et al., 2021 [17]	Consensus (SAM)	ED/ICU	Physiologically difficult airway	SAM consensus endorsing HOP framework and pre-intubation physiological optimization as standard of care
Miller et al., 2016 [18]	Prospective obs.	Prehospital	Ketamine RSI in shock	Patients with a great shock index had a blunted blood pressure response and were more likely to develop hypotension after ketamine induction
Tran et al., 2015 [19]	Cochrane SR	OR/ED	Rocuronium vs. succinylcholine	At 1.2 mg/kg, rocuronium produced intubating conditions comparable to succinylcholine, although succinylcholine was clinically superior for achieving excellent intubating conditions
Weingart and Levitan, 2012 [20]	Review	ED	Preoxygenation	Evidence supports apneic oxygenation via nasal cannula to extend the safe apneic window during RSI
Oliveira e Silva et al., 2017 [21]	SR and meta-analysis	ED/ICU	Apneic oxygenation	Apneic oxygenation was associated with reduced peri-intubation hypoxemia, higher first-pass success, and higher lowest peri-intubation oxygen saturation, but not with a significant reduction in severe hypoxemia
Prekker et al., 2023 [22]	RCT (NEJM)	ED/ICU	VL vs. DL in critically ill	DEVICE trial: VL improved FPS vs. DL (85.1% vs. 70.8%; 95% CI 9.9-18.7; p<0.001)
Chrimes, 2016 [23]	Conceptual report	ED/OR/ICU	Vortex approach	Universal CICO cognitive framework: reduces task fixation and delays in escalation to eFONA
Brown et al., 2015 [24]	Multicenter obs. (NEAR)	ED	VL across experience levels	In 17,583 ED intubations, overall first attempt success was 83% and increased from 80 to 86% over the study period, while video laryngoscope use increased substantially
Sakles et al., 2017 [25]	Prospective observational study	Academic ED	VL as primary vs. rescue	Predicted difficult airways occurred in 11% of non-arrest ED intubations; among difficult-airway patients receiving NMBA-facilitated intubation, video laryngoscopy was used in all cases, with 90% first-pass success
Price and McCoy, 2019 [26]	Educational review	ED/OR	Emergency FONA	Scalpel-bougie-tube eFONA as definitive CICO rescue; surgical anatomy and simulation training requirements
Gomez-Rios et al., 2025 [27]	Narrative review	Multidisciplinary	Human factors	Communication failures, leadership gaps, and poor situational awareness implicated in most preventable airway events
Fouche et al., 2014 [28]	SR and meta-analysis	Prehospital	Airways in OHCA	No clearly superior airway method in OHCA; equipoise between BVM and advanced airway persists

Included studies

Twenty-eight studies met the eligibility criteria and were included in the final synthesis. The included studies covered a broad range of designs: two randomized controlled trials, four systematic reviews and meta-analyses, three national or multicenter observational registry studies, seven prospective or retrospective cohort studies, one technical report, three clinical practice guidelines, five narrative and educational reviews, and three conceptual or consensus framework documents. This mix of study designs reflects the nature of emergency airway research, where large randomized trials are logistically difficult to conduct and much of the available evidence comes from registry data, observational studies, and expert consensus. Study characteristics for all included studies are summarized in Table 1.

The included studies were conducted across a wide range of clinical settings and geographic regions. Settings included EDs, intensive care units, prehospital services, and multi-institutional registries. Studies originated from the United States, United Kingdom, Europe, and Asia, providing a reasonably broad international perspective on emergency airway practice. However, the majority of included studies were conducted in well-resourced academic centers, which limits the generalizability of findings to lower-resource settings.

The included literature comprised a range of evidence types, including randomized controlled trials, systematic reviews and meta-analyses, observational studies, clinical practice guidelines, narrative reviews, and consensus or conceptual framework documents. Given the narrative nature of this review and the heterogeneity of the available evidence, the findings were synthesized descriptively, with consideration of the methodological strengths and limitations of the individual studies and evidence types.

Challenges in difficult airway assessment

The anatomical tools that emergency physicians have traditionally used to predict a difficult airway, with the Mallampati classification and thyromental distance being the most widely applied, were originally developed and validated in elective surgical patients. These are cooperative, fasted, pre-assessed patients who can sit upright, open their mouths on command, and hold still for examination. In the ED, none of these conditions can be assumed. The patient may be agitated, confused, or unconscious. A cervical collar might be in place. There may be blood or secretions obscuring the airway structures. The assessment has to happen in seconds, not minutes. Under these conditions, tools that performed reasonably well in the elective setting lose much of their predictive accuracy, as Reed demonstrated in a prospective ED study showing that standard anatomical scoring had limited discriminative value in emergency populations [10].

The look externally, evaluate 3-3-2, Mallampati, obstruction, neck mobility (LEMON) criteria (Table 2) offer a structured framework for bedside airway assessment that is more adaptable to emergency conditions than Mallampati alone. However, LEMON remains fundamentally an anatomical tool. It does not account for the physiological state of the patient, and in the ED, physiology may be a more important predictor of serious complications during intubation. The hypoxemia, extremes of size/age, anatomical abnormalities, vomiting/blood, exsanguination, neck movement (HEAVEN) criteria were developed specifically to address this gap (Table 3). HEAVEN incorporates Hypoxemia, Extremes of size/age, Anatomical abnormalities, Vomit/Blood, Exsanguination, and Neck mobility, integrating hemodynamic, metabolic, and environmental risk factors alongside anatomical considerations into a single risk stratification instrument suited to the emergency context [11,29]. This provides a more comprehensive assessment of emergency airway risk by integrating both anatomical and physiological factors. However, the HEAVEN criteria are intended to complement, rather than replace, conventional anatomical airway assessment, and further external validation is needed.

**Table 2:** The LEMON criteria for anatomical airway evaluation in the ED ED: emergency department; LEMON: look externally, evaluate 3-3-2, Mallampati, obstruction, neck mobility

Component	Description	Clinical application
Look externally	Physical characteristics	Facial trauma, beard, macroglossia, morbid obesity
Evaluate 3-3-2	Anatomical distances	Mouth opening ≥3 fingers; hyoid-mentum ≥3 fingers; thyroid-hyoid ≥2 fingers
Mallampati	Oropharyngeal view	Class I-IV; often impractical in uncooperative or obtunded patients
Obstruction	Physical blockages	Epiglottitis, peritonsillar abscess, angioedema, direct airway trauma
Neck mobility	Range of motion	Limited by cervical collar, degenerative joint disease, or traumatic injury

**Table 3:** The HEAVEN criteria for emergency airway risk assessment HEAVEN: hypoxemia, extremes of size/age, anatomical abnormalities, vomiting/blood, exsanguination, neck movement; SALAD: suction-assisted laryngoscopy and airway decontamination; SpO₂: peripheral oxygen saturation; PICA: peri-intubation cardiac arrest

Component	Parameter	Clinical significance
Hypoxemia	SpO₂ <95% on supplemental oxygen	Substantially curtails safe apnea time; markedly increases risk of PICA
Extremes of size/age	Morbid obesity; pediatric patients	Altered airway anatomy and physiology; reduced functional residual capacity
Anatomical abnormalities	Distorted landmarks	Trauma, masses, burns, or prior surgery altering glottic anatomy
Vomit/blood	Airway contamination	Obscures glottic view; mandates aggressive suctioning (SALAD technique)
Exsanguination	Hemorrhagic shock/severe hypotension	Markedly elevated risk of peri-intubation hemodynamic collapse
Neck mobility	Limited range of motion	Prevents optimal sniffing position due to collar, injury, or degenerative disease

Tan et al. evaluated the HEAVEN criteria prospectively in 174 emergency intubations. The presence of any HEAVEN criterion was associated with reduced first-pass success, and physiological criteria, particularly hypoxemia and vomit/blood/fluid, were associated with intubation complications, although the anatomical-challenge criterion was the only individual predictor of first-pass success [11]. These findings support HEAVEN as a promising tool that captures physiological risk beyond anatomy while confirming that further validation is needed.

Children present their own distinct set of airway challenges that deserve specific attention. Several anatomical differences make pediatric airway management more demanding than adult intubation. The occiput is proportionately larger, which causes passive neck flexion when the child lies flat, the opposite of the position needed for laryngoscopy. The larynx sits higher and farther forward in the neck. The tongue is relatively large for the size of the oral cavity. The epiglottis is omega-shaped rather than flat, making it harder to lift with a standard blade. Taken together, these features make obtaining a clear glottic view more technically challenging in children than in adults. On top of these factors, children have significantly smaller oxygen reserves than adults; functional residual capacity is lower, and oxygen consumption per kilogram is higher. This means that desaturation during apnea happens faster and more severely in a child than in an adult with the same clinical condition. Drug dosing adds another layer of complexity; induction agents and neuromuscular blocking drugs must be calculated by weight. Registry data from Kerrey et al. confirm that failed intubation attempts and adverse events occur more frequently during pediatric emergency RSI than in adults and emphasize the necessity of pediatric-specific institutional protocols [12].

The role of point-of-care ultrasound in airway assessment

Point-of-care ultrasonography (POCUS) has become an increasingly useful addition to clinical bedside airway assessment in the ED [13]. Its most important practical advantage over clinical scoring systems is that it does not depend on patient cooperation. A Mallampati assessment requires the patient to sit upright, open their mouth, and protrude their tongue on command. In an agitated, confused, or critically ill patient, this procedure is simply not possible. An important practical limitation of POCUS in this context is that it is operator-dependent and requires structured training and regular practice to achieve reliable results. EDs should ensure that clinicians performing airway POCUS have received appropriate training and that competency is maintained through simulation-based education.

Beyond its practical advantages, POCUS provides objective, measurable anatomical information that can improve the prediction of difficult laryngoscopy, especially in the ED. Sonographic measurements, including the distance from the skin to the epiglottis and anterior soft-tissue thickness at the level of the hyoid bone, have shown better predictive accuracy for difficult intubation than clinical assessment alone. A systematic review by Gottlieb et al. confirmed that POCUS-based pre-intubation assessment improved risk stratification accuracy across both ED and ICU settings [13]. In obese patients in particular, in whom neck anatomy is difficult to assess by palpation and Mallampati grading is unreliable, ultrasound provides information that clinical examination simply cannot.

One of the most clinically important and consistently underused applications of POCUS in airway management is preprocedural identification of the cricothyroid membrane (CTM). In patients with obesity, a short muscular neck, cervical edema, or previous neck surgery, finding the CTM by feel alone is unreliable and often impossible. This information matters enormously when a patient cannot be intubated or oxygenated (CICO) and a scenario develops in which every second matters; the clinician needs to know exactly where to place the scalpel. Marking the CTM with ultrasound before induction, using either the laryngeal handshake technique or the thyroid cartilage, airway, cricoid cartilage, airway (TACA) scanning approach (thyroid cartilage, airway, cricoid cartilage, and airway), takes less than a minute and substantially reduces this uncertainty. The DAS 2025 guidelines recommend pre-identification and, where appropriate, marking of the CTM in patients with anticipated difficult airways to facilitate timely emergency front-of-neck airway (eFONA) access if required. Although this simple step is easy to overlook, pre-identifying the CTM before induction can be life-saving when eFONA access is required; failure to do so may delay airway access at a critical moment, with potentially life-threatening consequences [30].

Point-of-care ultrasound may provide rapid adjunctive confirmation of endotracheal tube position, particularly when auscultation is unreliable or capnographic interpretation is delayed or physiologically limited. Transtracheal ultrasound can rapidly identify tracheal versus esophageal placement: tracheal intubation is identified by a thin hyperechoic membrane just posterior to the trachea, whereas esophageal intubation demonstrates the characteristic double-tract sign [13]. Lung sliding and diaphragmatic motion provide indirect evidence of ventilation and may help detect endobronchial malposition. Waveform end-tidal CO₂ monitoring remains the standard method for confirming tube placement, with POCUS serving as a valuable adjunct when conventional confirmation is delayed, unreliable, or insufficient.

The physiologically difficult airway

Hemodynamic Instability and Positive Pressure Ventilation

Induction and initiation of positive-pressure ventilation can substantially alter cardiovascular physiology in critically ill patients [14]. Induction agents reduce catecholamine-mediated vascular tone, producing vasodilation and blunting myocardial contractility. When PPV commences, it causes a rise in intrathoracic pressure, compresses the major veins, and reduces venous return. In a patient who is already in septic or hemorrhagic shock and requiring adrenaline to maintain a marginal blood pressure, this combination can tip the patient into cardiac arrest within seconds of intubation. This is PICA, which occurs in roughly 1% to 4% of emergency intubations [4]. Failure to anticipate peri-intubation hemodynamic deterioration may increase the risk of preventable cardiovascular collapse.

Push-dose vasopressors, such as small bolus doses of epinephrine or phenylephrine given intravenously, can raise mean arterial pressure quickly and buy time to intubate more safely. Singer et al. showed that push-dose epinephrine and phenylephrine are safe and effective in critically ill adults, producing clinically meaningful blood pressure improvement without significant adverse effects [15]. Practical targets and sequencing for this optimization are detailed in the HOP framework.

Metabolic Acidosis and the Apneic Window

Severe metabolic acidosis creates a specific and underappreciated danger during emergency intubation that has nothing to do with airway anatomy. Consider a patient with diabetic ketoacidosis or severe salicylate toxicity who is breathing rapidly with deep respirations because this compensatory hyperventilation is essential to maintain physiological compensation for the underlying metabolic acidosis. The moment a neuromuscular blocking agent is administered during RSI and the patient stops breathing, that compensatory hyperventilation stops with it. Carbon dioxide accumulates rapidly. Even brief periods of apnea can cause rapid carbon dioxide accumulation and a precipitous fall in pH, potentially resulting in hemodynamic deterioration or cardiac arrest [16]. A clinician focused purely on anatomical airway assessment, mouth opening, neck mobility, and Mallampati grade will miss this entirely. It will not show up on any scoring tool. But it can kill the patient during what appears to be a straightforward intubation.

In patients in whom this risk is recognized, DSI with ketamine offers a practical alternative to standard RSI [1]. Rather than giving the full induction sequence immediately, ketamine is used to sedate the patient enough to tolerate preoxygenation, allowing oxygen saturation to be optimized and the apneic window to be extended while spontaneous breathing is maintained. The paralytic agent is given only once preoxygenation is adequate. This approach is clinically logical and has shown promising results in observational studies. However, the evidence base for DSI remains largely single-center and observational; no large randomized controlled trial has compared DSI directly with standard RSI. Patient selection is important. DSI is best suited for agitated or hypoxic patients who cannot tolerate preoxygenation. It should not be used in patients with impending respiratory arrest, in whom immediate airway management is required.

The Hypotension, Oxygenation, and pH Framework: Resuscitation Before Intubation

Weingart originally described a structured pre-intubation optimization framework that was subsequently formalized in the Society for Airway Management consensus by Kornas et al. Summarized by the mnemonic HOP (Hypotension, Oxygenation, and pH), it identifies three physiological targets that must be addressed before laryngoscopy in any unstable patient (Table 4). The strength of HOP is not its complexity but the discipline it enforces, requiring the clinician to ask three specific questions before reaching for the laryngoscope: Is the blood pressure adequate? Is oxygenation optimized? And is there a dangerous metabolic acidosis that needs to be accounted for before apnea is induced [17]?

**Table 4:** The HOP framework: key physiological targets for pre-intubation optimization HOP: hypotension-oxygenation-pH; MAP: mean arterial pressure; NIPPV: non-invasive positive pressure ventilation; SpO₂: peripheral oxygen saturation

Component	Physiological target	Clinical intervention
H-hypotension	MAP sufficient to tolerate induction	Judicious fluid resuscitation and/or push-dose vasopressors prior to laryngoscopy
O-oxygenation	SpO₂ optimization to maximize safe apneic time	High-flow O₂ (≥15 L/min) via non-rebreather mask; apneic oxygenation via nasal cannula; NIPPV in selected patients
P-pH/Acidosis	Prevention of critical pH deterioration during apneic window	Recognize compensatory hyperventilation dependency; match pre-induction minute ventilation during peri-intubation period

The H addresses hypotension: A patient who is in shock and intubated without prior hemodynamic optimization is at significant risk of cardiac arrest during or immediately after induction. The practical target is a MAP of at least 65 mmHg before induction, achieved through fluid resuscitation and push-dose vasopressors if needed. However, MAP targets should be individualized in patients with conditions such as chronic hypertension or traumatic brain injury, where higher perfusion pressures may be clinically appropriate. These interventions should be initiated before laryngoscopy rather than as a rescue after hemodynamic collapse.

The O addresses oxygenation: preoxygenation and apneic oxygenation, detailed above, should be maximized before induction so that the safe apnea window is as long as possible. A saturation that does not respond to these measures is a reason to pause, not to proceed faster.

The P addresses pH: paralysis abruptly removes the compensatory hyperventilation that patients with severe metabolic acidosis depend on (discussed above). The response is not to correct the acidosis before intubating, as there is rarely time, but to recognize this dependency and match the patient’s pre-induction respiratory rate and tidal volume during and after the procedure to prevent pH collapse.

Together, these three steps reframe emergency intubation as a resuscitative sequence rather than a standalone procedure. The time before induction in an unstable patient should be used to optimize physiology as much as possible by improving blood pressure, maximizing oxygenation, and identifying metabolic risks. Although complete stabilization is often not possible in the ED, these measures help ensure the patient is in the best possible condition to tolerate intubation. However, this optimization should never delay definitive airway management in patients with immediately life-threatening airway compromise, cardiac arrest, or crash airway situations, where rapid airway intervention is essential.

Pharmacological strategies in the unstable patient

Induction Agents: Ketamine Versus Etomidate

Selection of an induction agent in the ED should be individualized according to the patient's current hemodynamic status, underlying pathology, and physiological reserve. The same drug that is safe and appropriate in a normotensive patient can cause cardiovascular collapse in someone who is already in shock. Drug selection must therefore be matched to the individual patient, their hemodynamic state, their underlying condition, and their remaining physiological reserve.

Etomidate has been the most widely used induction agent for emergency airway management. Its hemodynamic neutrality and minimal effects on heart rate, systemic vascular resistance, and myocardial contractility make it a relatively safe choice when the hemodynamic consequences of other agents are a concern. In patients with uncertain or borderline hemodynamic status, etomidate is often the induction agent of choice because it is less likely to worsen hemodynamic instability during intubation. The one caveat that emergency physicians need to be aware of is etomidate's effect on the adrenal gland. It suppresses cortisol production by inhibiting 11β-hydroxylase, and this effect is most relevant in patients with septic shock, who are both the most common group to receive etomidate in the ED and the group most likely to have stress-related adrenal insufficiency already. Current evidence does not show a clear survival disadvantage from a single induction dose of etomidate in sepsis, but this remains an active area of debate and should factor into decision-making in individual patients.

Ketamine has become the preferred induction agent in several specific clinical situations. In patients with severe asthma or chronic obstructive pulmonary disease (COPD), its bronchodilatory properties make it particularly useful; it relaxes airway smooth muscle at the same time as it induces anesthesia. In distributive shock, ketamine stimulates endogenous catecholamine release, which helps maintain blood pressure during induction. Miller et al. demonstrated favorable hemodynamic responses to ketamine-based RSI in prehospital patients at risk of shock [18]. One important limitation deserves mention: in patients who are in profound or prolonged shock and have already exhausted their catecholamine reserves, ketamine's direct negative inotropic effect can paradoxically worsen hemodynamics rather than improve them. This phenomenon is uncommon but real, and clinicians need to be aware of it. Propofol should be avoided in patients with hemodynamic compromise, as it causes significant vasodilation and myocardial depression even in relatively stable patients, and its effects in a shocked patient can be severe and rapid.

Neuromuscular Blocking Agents: Rocuronium Versus Succinylcholine

Neuromuscular blockade practice in emergency RSI has moved substantially toward rocuronium over the past decade. Succinylcholine has a very short duration of action, which is theoretically attractive if intubation fails; the paralysis wears off relatively quickly. In practice, however, succinylcholine carries several contraindications that come up regularly in ED patients. It causes dangerous hyperkalemia in patients with renal failure, rhabdomyolysis, crush injuries, burns, or prolonged immobility, conditions that are not uncommon in emergency presentations. Rocuronium at 1.2 mg/kg provides intubating conditions equivalent to those of succinylcholine without this risk, as confirmed by the Cochrane review by Tran et al. [19]. Its longer duration of action is also practically useful; it reduces patient-ventilator fighting in the post-intubation period and gives the team more time to manage a difficult or failed attempt. The one requirement when using rocuronium is ensuring that sugammadex is immediately available, as it reverses rocuronium rapidly and completely and is the essential backup if intubation fails and the paralysis needs to be reversed urgently.

Airway preparation

The Emergency Department Airway Bundle

The airway bundle is based on a straightforward observation: intubation in the ED is not a single moment; it is a sequence of steps, and complications can arise at any point in that sequence. Treating it as a staged procedure with a preparation phase, a procedural phase, and a post-intubation phase, rather than simply picking up a laryngoscope and intubating, significantly reduces the risk of serious adverse events. Jaber et al. provided the clearest evidence for this in a prospective multicenter study showing that a structured pre-intubation checklist and post-intubation monitoring protocol significantly reduced life-threatening complications in critically ill patients [9]. The DAS 2018 guidelines for critically ill adults further endorsed bundle-based care as standard practice [8]. Essential components of the recommended bundle are outlined in Table 5.

**Table 5:** The ED airway bundle, equipment, and preparation checklist Preoxygenation strategy supported by Weingart and Levitan [20]. eFONA: emergency front-of-neck access; CTM: cricothyroid membrane; LMA: laryngeal mask airway; POCUS: point-of-care ultrasound; IV: intravenous; ED: emergency department; CO₂: carbon dioxide

Category	Essential components
Preparation	Dual Yankauer suction, waveform end-tidal CO₂ monitoring, dual IV access, pre-drawn vasopressors and sugammadex
Preoxygenation	High-flow O₂ (≥15 L/min) via non-rebreather mask plus nasal cannula for continuous apneic oxygenation [20]
Primary device	Video laryngoscope as first-line device; bougie immediately available
Backup/rescue device	Second-generation supraglottic airway (e.g., i-gel or LMA supreme)
eFONA kit	Scalpel-bougie-tube cricothyrotomy set with CTM pre-identified by POCUS before induction

Preoxygenation is the single most important preparatory step before emergency intubation. The goal is very simple: to fill the patient's oxygen reserves as completely as possible before apnea begins, so that the clinician has the maximum amount of time to intubate before dangerous desaturation occurs. In practice, this means high-flow oxygen at 15 L/min or more via a well-fitting non-rebreather mask, started as early as possible and continued right up to the moment of induction [20]. In addition, apneic oxygenation via nasal cannula, keeping low-flow oxygen running through the nose throughout the apneic period, has been shown to meaningfully extend the safe apneic window. Oliveira e Silva et al. confirmed in a systematic review that using apneic oxygenation during intubation reduces peri-intubation hypoxemia [21]. Post-intubation, immediate reassessment of tube position, oxygenation, ventilation, and blood pressure should follow as standard practice. In patients who are too hypoxic to preoxygenate adequately with a non-rebreather mask alone, noninvasive positive-pressure ventilation can be used before induction to improve baseline oxygen saturation. This is the principle behind DSI [1].

First-pass intubation success is the most important performance metric in emergency airway management, and the reason is straightforward. Every failed laryngoscopy attempt makes the situation worse. Oxygen saturation falls further, blood pressure drops, the airway becomes more traumatized and swollen, and the risk of cardiac arrest rises with each additional attempt [4]. Getting it right the first time is not just a quality measure; it is a patient safety imperative. The strongest evidence on how to achieve this comes from the direct versus video laryngoscope (DEVICE) trial, a multicenter randomized controlled trial enrolling 1,417 critically ill adults across 17 ED and ICU sites in the United States [22]. It showed that video laryngoscopy improved first-pass success from 70.8% with direct laryngoscopy to 85.1%, a difference of 14.3 percentage points that is both statistically robust and clinically meaningful. Registry data from Brown et al. demonstrated high overall first-attempt success in ED intubations, while Sakles et al. reported 90% first-pass success when video laryngoscopy was used for neuromuscular-blocking-agent-facilitated intubation in patients with predicted difficult airways [24,25].

The advantage of video laryngoscopy goes beyond the improvement in first-pass success rates. When a video laryngoscope is used, everyone in the room can see the glottis on the screen: the intubating physician, the assistant, the team leader, and the nurse drawing up drugs. This shared view changes the dynamic of the procedure fundamentally. The assistant can see exactly what the laryngoscopist sees and can provide meaningful real-time guidance. The team leader can immediately identify if the tube is going in the wrong direction. Unrecognized esophageal intubation is a catastrophic and preventable complication that becomes far less likely when four pairs of eyes are watching the same image. In practical terms, video laryngoscopy turns what has traditionally been a solo technical procedure into a team effort. Emergency physicians should be proficient with both video and direct laryngoscopy. There are situations where direct laryngoscopy remains appropriate, but video laryngoscopy should be the default first choice in the ED.

Rescue Strategies and Front-of-Neck Access

One of the biggest dangers during airway management is continuing the same technique even when it is not working. In a stressful emergency, clinicians may become focused on repeated intubation attempts and lose valuable time. This can eventually lead to a “can’t intubate, can’t oxygenate” (CICO) situation. The Vortex Approach helps prevent this problem by providing a simple and easy-to-remember airway strategy. It focuses on three non-surgical airway options: facemask ventilation, supraglottic airway insertion, and endotracheal intubation. Each technique should be given the best possible chance of success before moving to the next option. If all three methods fail to provide adequate oxygenation despite proper optimization, an eFONA should be performed without delay [23].

Although cricothyrotomy is a life-saving procedure, most emergency physicians perform it very rarely. Because of this, regular simulation training is important to maintain skills and confidence. Airway teams should also ensure that a dedicated scalpel-bougie-tube cricothyrotomy kit is immediately available whenever advanced airway management is planned [26]. The standard technique involves a horizontal stab incision through the CTM, bougie insertion into the trachea, and a cuffed endotracheal tube railroaded over the bougie, a sequence that takes under 30 seconds in practiced hands. Current airway guidelines from the DAS and the ASA recommend identifying the CTM before intubation, especially in patients with a potentially difficult airway, and stress that every airway plan should include a clear surgical airway rescue strategy [7,8]. In out-of-hospital cardiac arrest, no single airway method has been shown to be clearly superior, and equipoise persists between bag-valve-mask ventilation and advanced airway placement [28].

Human factors, team dynamics, and post-intubation care

Successful emergency airway management depends not only on technical skills but also on effective teamwork and communication. During emergency intubation, clinicians must make rapid decisions while coordinating multiple tasks simultaneously. Studies have shown that poor communication, unclear leadership, and loss of situational awareness contribute significantly to airway-related adverse events [27]. To reduce these risks, many EDs use a brief pre-intubation team discussion or “10-second huddle,” during which the team reviews the primary plan, backup airway strategy, rescue plan, and individual roles. Difficult-airway guidelines emphasize limiting repeated airway attempts, reassessing oxygenation and ventilation, and changing the airway strategy when initial attempts are unsuccessful [7,8].

PICA, although uncommon, remains a serious complication of emergency airway management. Current evidence supports a resuscitation-focused approach that includes optimizing hemodynamics before induction, using apneic oxygenation with a high-flow nasal cannula during intubation to reduce severe desaturation, and performing immediate post-intubation reassessment of tube position, oxygenation, ventilation, and blood pressure [21]. Early recognition and correction of hemodynamic instability can help prevent further deterioration after intubation.

Limitations of existing evidence

The evidence supporting current emergency airway practice is meaningful but has real limitations. Most available data come from large observational registries, primarily the National Emergency Airway Registry and equivalent national databases, rather than randomized controlled trials. Registry data are vulnerable to selection bias, inconsistent outcome reporting across institutions, and wide variation in operator experience, local protocols, and available equipment. The clinical diversity of ED patients means findings from one population may not apply straightforwardly to another.

Most emergency airway studies measure process outcomes rather than patient outcomes. First-pass success is a useful proxy for safety, but what ultimately matters is whether the patient survives with good neurological function. However, long-term neurological recovery has not been consistently reported in the available literature. Large randomized trials in critically ill patients are genuinely difficult to conduct, but surrogate outcomes alone cannot answer the questions that matter most clinically.

Most research included in this review was conducted in well-resourced academic EDs in high-income countries where video laryngoscopes, POCUS, sugammadex, and structured airway teams are standard. These resources are not available everywhere. The applicability of these findings to community hospitals, rural EDs, and EDs in low- and middle-income countries remains uncertain and largely untested. This is not a minor limitation; the majority of emergency intubations worldwide happen outside academic centers, and those patients have the most to gain from evidence-based protocols.

We acknowledge the limitations of this review itself. Narrative reviews involve interpretive judgment that preregistered systematic reviews minimize. The findings should therefore be interpreted as an evidence-informed clinical synthesis rather than as meta-analytically derived estimates. Machine learning-based airway prediction tools and AI-assisted clinical decision support show early promise but have not yet been prospectively validated in real-world emergency settings. They are worth watching but are not yet ready for routine clinical use.

Evidence-based clinical recommendations

Across the included studies, a consistent message emerges: emergency intubation managed using a physiology-based approach appeared to be associated with better outcomes than intubation treated as a standalone technical procedure. The practical recommendations that follow are summarized in Table 6. They are not a rigid protocol; they are a framework to be adapted to the individual patient, clinical setting, and available resources.

**Table 6:** Evidence-based practice recommendations for emergency airway management Recommendation strength was assigned by author consensus: "Strong" for recommendations supported by randomized controlled trials or consistent systematic-review evidence, and "Moderate" for those based on observational studies, clinical practice guidelines, or expert consensus. FPS: first-pass success; MAP: mean arterial pressure; SGA: supraglottic airway; CICO: cannot intubate, cannot oxygenate; ETCO₂: end-tidal carbon dioxide; POCUS: point-of-care ultrasound; RSI: rapid sequence intubation; SAM: Society for Airway Management; CTM: cricothyroid membrane; HEAVEN: hypoxemia, extremes of size/age, anatomical abnormalities, vomiting/blood, exsanguination, neck movement

Recommendation	Evidence base	Strength
Apply HEAVEN criteria for pre-intubation physiological risk stratification	Tan et al. [11]; Mosier et al. [14]	Moderate
Optimize hemodynamic pre-intubation with push-dose vasopressors when MAP is inadequate	Singer et al. [15]; Kornas et al. [17]	Moderate-strong
Use high-flow apneic oxygenation via nasal cannula throughout RSI	Oliveira e Silva et al. [21]	Strong
Select video laryngoscopy as the primary intubation device	Prekker et al. [22]; Brown et al. [24]	Strong
Pre-identify CTM with POCUS in anticipated difficult airways	Gottlieb et al. [13]	Moderate
Conduct a structured 10-second pre-intubation team briefing before every induction	Gómez-Ríos et al. [27]; Cook et al. [3]	Moderate
Limit laryngoscopic attempts to two before changing device or escalating to SGA	Kornas et al. [17]; Walls et al. [4]	Moderate-strong
Implement the Vortex Approach as the institutional cognitive aid for CICO scenarios	Chrimes [23]; Price and McCoy [26]	Moderate
Confirm tube placement immediately after intubation with waveform ETCO₂ whenever available, and consider POCUS as an adjunct in trained hands, especially when auscultation is unreliable, capnography is delayed or physiologically limited, or endobronchial placement is suspected	Gottlieb et al. [13]; Apfelbaum et al. [7]	Strong
Implement a structured departmental airway bundle protocol	Jaber et al. [9]; Higgs et al. [8]	Moderate-strong

**Figure 1:**
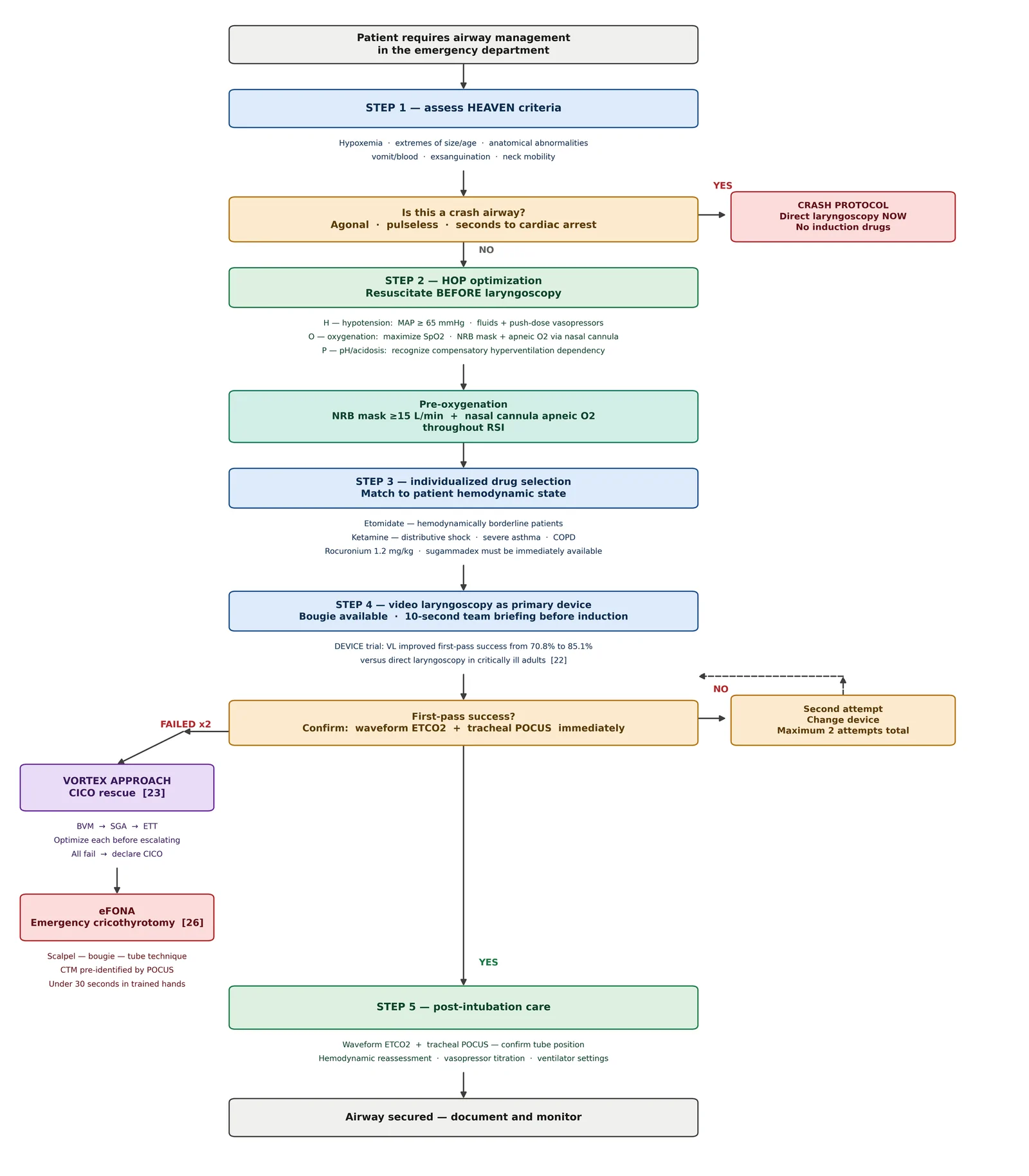
Clinical decision algorithm for emergency airway management HEAVEN: Hypoxemia, Extremes of size/age, Anatomical abnormalities, Vomit/Blood, Exsanguination, Neck mobility; HOP: Hypotension, Oxygenation, pH; NRB: non-rebreather mask; MAP: mean arterial pressure; SpO₂: peripheral oxygen saturation; RSI: rapid sequence intubation; ETCO₂: end-tidal carbon dioxide; POCUS: point-of-care ultrasound; BVM: bag-valve-mask; SGA: supraglottic airway; ETT: endotracheal tube; eFONA: emergency front-of-neck access; CTM: cricothyroid membrane; CICO: cannot intubate, cannot oxygenate; COPD: chronic obstructive pulmonary disease; VL: video laryngoscopy The authors take full responsibility for the scientific accuracy, content, and integrity of the figure. Image credit: Anagani Hrushikesh, created using Claude AI (Anthropic PBC, San Francisco, CA, USA).

Several recommendations are supported by relatively strong evidence, including video laryngoscopy as the primary device in many emergency airway settings and apneic oxygenation during RSI. Immediate post-intubation confirmation should include waveform capnography whenever available, while POCUS may serve as a rapid adjunct, particularly when conventional methods are delayed, unreliable, or insufficient to evaluate endobronchial malposition. Others, including the HEAVEN criteria and the Vortex Approach, are supported by moderate-quality evidence and expert consensus, representing the best current guidance where large randomized trials remain difficult to conduct. A clinical decision algorithm summarizing this physiology-first approach to emergency airway management is presented in Figure 1.

## Conclusions

Emergency airway management has evolved from an anatomy-focused assessment to a physiology-first, systems-based resuscitative approach. Physiological instability, particularly hypoxemia, shock, and metabolic acidosis, has consistently been associated with an increased risk of peri-intubation complications. Optimal emergency airway management requires integration of both anatomical assessment and physiological optimization, as these complementary approaches contribute to comprehensive airway risk assessment and safer intubation.

Current evidence supports a structured approach to emergency airway management that includes pre-intubation physiological optimization using the HOP framework, video laryngoscopy, apneic oxygenation, individualized drug selection, and a preplanned surgical rescue pathway. The DEVICE trial, together with emerging evidence supporting POCUS integration and cognitive tools such as the Vortex Approach, has further strengthened the evidence supporting this approach. The strength of evidence varies across these components, however, and implementation should account for local resources, operator training, and patient-specific physiology. Wider implementation across diverse and lower-resource emergency care settings, alongside further prospective studies evaluating patient-centered outcomes, including neurological recovery, remains an important priority for future research.

## References

[1] Weingart SD (2011). Preoxygenation, reoxygenation, and delayed sequence intubation in the emergency department. J Emerg Med.

[2] Lentz S, Grossman A, Koyfman A, Long B (2020). High-risk airway management in the emergency department. Part I: diseases and approaches. J Emerg Med.

[3] Cook TM, Woodall N, Harper J, Benger J (2011). Major complications of airway management in the UK: results of the fourth National Audit Project of the Royal College of Anaesthetists and the Difficult Airway Society. Part 2: Intensive care and emergency departments. Br J Anaesth.

[4] Walls RM, Brown CA III, Bair AE, Pallin DJ (2011). Emergency airway management: a multi-center report of 8937 emergency department intubations. J Emerg Med.

[5] Driver BE, Prekker ME, Klein LR (2018). Effect of use of a bougie vs endotracheal tube and stylet on first-attempt intubation success among patients with difficult airways undergoing emergency intubation: a randomized clinical trial. J Am Med Assoc.

[6] Root CW, Mitchell OJ, Brown R (2020). Suction Assisted Laryngoscopy and Airway Decontamination (SALAD): a technique for improved emergency airway management. Resusc Plus.

[7] Apfelbaum JL, Hagberg CA, Connis RT (2022). 2022 American Society of Anesthesiologists practice guidelines for management of the difficult airway. Anesthesiology.

[8] Higgs A, McGrath BA, Goddard C, Rangasami J, Suntharalingam G, Gale R, Cook TM (2018). Guidelines for the management of tracheal intubation in critically ill adults. Br J Anaesth.

[9] Jaber S, Jung B, Corne P (2010). An intervention to decrease complications related to endotracheal intubation in the intensive care unit: a prospective, multiple-center study. Intensive Care Med.

[10] Reed MJ, Dunn MJ, McKeown DW (2005). Can an airway assessment score predict difficulty at intubation in the emergency department?. Emerg Med J.

[11] Tan NE, Yoong KP, Yahya HM (2022). Use of HEAVEN criteria for predicting difficult intubation in the emergency department. Clin Exp Emerg Med.

[12] Kerrey BT, Rinderknecht AS, Geis GL, Nigrovic LE, Mittiga MR (2012). Rapid sequence intubation for pediatric emergency patients: higher frequency of failed attempts and adverse effects found by video review. Ann Emerg Med.

[13] Gottlieb M, O'Brien JR, Ferrigno N, Sundaram T (2024). Point-of-care ultrasound for airway management in the emergency and critical care setting. Clin Exp Emerg Med.

[14] Mosier JM, Joshi R, Hypes C, Pacheco G, Valenzuela T, Sakles JC (2015). The physiologically difficult airway. West J Emerg Med.

[15] Singer S, Pope H, Fuller BM, Gibson G (2022). The safety and efficacy of push dose vasopressors in critically ill adults. Am J Emerg Med.

[16] Kraut JA, Madias NE (2010). Metabolic acidosis: pathophysiology, diagnosis and management. Nat Rev Nephrol.

[17] Kornas RL, Owyang CG, Sakles JC, Foley LJ, Mosier JM (2021). Evaluation and management of the physiologically difficult airway: consensus recommendations from society for Airway Management. Anesth Analg.

[18] Miller M, Kruit N, Heldreich C, Ware S, Habig K, Reid C, Burns B (2016). Hemodynamic response after rapid sequence induction with ketamine in out-of-hospital patients at risk of shock as defined by the shock Index. Ann Emerg Med.

[19] Tran DT, Newton EK, Mount VA, Lee JS, Wells GA, Perry JJ (2015). Rocuronium versus succinylcholine for rapid sequence induction intubation. Cochrane Database Syst Rev.

[20] Weingart SD, Levitan RM (2012). Preoxygenation and prevention of desaturation during emergency airway management. Ann Emerg Med.

[21] Oliveira J E Silva L, Cabrera D, Barrionuevo P, Johnson RL, Erwin PJ, Murad MH, Bellolio MF (2017). Effectiveness of apneic oxygenation during intubation: a systematic review and meta-analysis. Ann Emerg Med.

[22] Prekker ME, Driver BE, Trent SA (2023). Video versus direct laryngoscopy for tracheal intubation of critically ill adults. N Engl J Med.

[23] Chrimes N (2016). The Vortex: a universal 'high-acuity implementation tool' for emergency airway management. Br J Anaesth.

[24] Brown CA III, Bair AE, Pallin DJ, Walls RM (2015). Techniques, success, and adverse events of emergency department adult intubations. Ann Emerg Med.

[25] Sakles JC, Douglas MJK, Hypes CD, Patanwala AE, Mosier JM (2017). Management of patients with predicted difficult airways in an academic emergency department. J Emerg Med.

[26] Price TM, McCoy EP (2019). Emergency front of neck access in airway management. BJA Educ.

[27] Gómez-Ríos MÁ, Michalek P, Gaszyński T, Van Zundert AA (2025). Human factors in airway management: designing systems for safer, team-based care. J Clin Med.

[28] Fouche PF, Simpson PM, Bendall J, Thomas RE, Cone DC, Doi SA (2014). Airways in out-of-hospital cardiac arrest: systematic review and meta-analysis. Prehosp Emerg Care.

[29] Kuzmack E, Inglis T, Olvera D, Wolfe A, Seng K, Davis D (2018). A novel difficult-airway prediction tool for emergency airway management: validation of the Heaven criteria in a large air medical cohort. J Emerg Med.

[30] Ahmad I, El-Boghdadly K, Iliff H (2026). Difficult Airway Society 2025 guidelines for management of unanticipated difficult tracheal intubation in adults. Br J Anaesth.

